# Physical Activity and Physical Fitness of Adults with Intellectual Disabilities in Group Homes in Hong Kong

**DOI:** 10.3390/ijerph15071370

**Published:** 2018-06-29

**Authors:** Bik C. Chow, Peggy H. N. Choi, Wendy Y. J. Huang

**Affiliations:** 1 Department of Physical Education, Hong Kong Baptist University, Kowloon Tong, Hong Kong; wendyhuang@hkbu.edu.hk; 2 Department of Sports and Recreation Management, Technological and Higher Education Institute of Hong Kong, Chai Wan, Hong Kong; pchoi@vtc.edu.hk

**Keywords:** physical activity, physical fitness, adults, intellectual disabilities, group home

## Abstract

Adults with intellectual disabilities (ID) typically have a sedentary lifestyle and higher rates of overweight and obesity. This study describes the habitual daily physical activity (PA) and the health-related physical fitness (PF) of adults with mild and moderate ID who resided in four group homes and worked in sheltered workshops. We also assessed the contribution of PF variables towards PA levels and sedentary behavior of this population subgroup. Adults with mild and moderate ID (*N* = 114) were assessed on PF tests (percent body fat, waist and hip circumferences, 6-min walk (6MWT), arm curl, and sit and reach). PA and sedentary behavior on weekdays were determined using Actigraph accelerometers. Results showed these adults averaged 2% of their daily time (or 10 min) engaged in moderate-to-vigorous PA (MVPA) and 67% of the time (495 min) being sedentary. No significant differences between mild and moderate ID were found for any PA or PF variable. Linear multiple regression analyses showed 6MWT to be the only significant PF variable contributing to the variance of PA and sedentary behavior. In conclusion, adults with ID reside in group home have low PA and low fitness levels. Among fitness variables, the walking test (i.e., cardiovascular fitness) had the highest positive association with participants’ daily PA, MVPA, and negative association with sedentary behavior. Future intervention studies in promoting PA and fitness for adults with ID are warranted.

## 1. Introduction

Intellectual disability (ID) is a disability characterized by significant limitations, both in intellectual functioning and in adaptive behavior, that begins before adulthood [1]. In Hong Kong, individuals with ID are classified into mild, moderate, severe, or profound grades through clinical assessment before the age of 18. In January 2015, there were 71,000–101,000 people with ID in Hong Kong, approximately 1.0–1.4% of the population [2]. This figure is similar to the estimated prevalence rate for high-income countries (0.9%) [3].

Adults with ID have been associated with having multiple health conditions and tend to have higher morbidity rates and a shorter life expectancy [4,5,6] than the general population. Nonetheless, the lifespans of adults with ID have increased over recent decades [7]. As people with ID often experience health problems associated with aging at earlier ages and at higher rates, researchers have stressed the importance of health promotion interventions for this population subgroup [8,9]. Increasing physical activity (PA), because of its proven association with favorable health outcomes (e.g., reduced risk of heart diseases, hypertension, cancer, diabetes, and obesity), is a recommended intervention/tactic [10]. The World Health Organization (WHO) (2010) recommends that adults aged 18–64 should do at least 150 min of moderate-intensity PA throughout the week [11]. Meanwhile, most studies of adults with ID show they have very low PA levels and have a sedentary lifestyle. Review has shown that only 9% [12] of adults with ID met the PA guidelines based on PA data from objective and subjective measures compared with 30% to 47% of the general population [13].

In addition to engaging in limited PA, adults with ID tend to have low cardiovascular fitness that starts at young age and deteriorates with age [14]. Additionally, compared to the general population, they tend to perform poorly on physical fitness tests [15,16]. A longitudinal study of physical fitness (PF) showed a greater decline over 13 years for adults with ID than with those without ID. There were greater increases in body mass and percentage of body fat in males and females with ID and there was greater decline in cardiovascular fitness and sit-ups in the females [17].

On the other hand, individuals with ID that are only capable of basic self-care who lack adequate daily living skills to live independently in the community may apply to live in the group homes [18]. Therefore, the group home is an important setting for individuals with ID which provides 24-h care and support. However, it is a restrictive environment in which residents have to obtain permission to go out from the home and this may reduce opportunities for PA and maintaining PF. There are currently 37 group homes offering 2092 residential occupancy for adults with mild and moderate ID in different districts in Hong Kong [18]. The majority of the group homes (78%, 1744 residents) are paired with sheltered workshops that are located nearby or in the same buildings. Sheltered workshops provide full-time employment for individuals with mild and moderate ID after their completion of education in the special schools at the age of 15 years old.

To our knowledge, there are very limited number of studies (*N* = 2) related to the PA of Hong Kong adults with ID and no study has been conducted on both PA and PF in this special population in Hong Kong. Chan and Chow [19] examined the PA of 30 adults with mild to moderate ID from a group home in Hong Kong. The residents were asked to wear pedometers for recording PA in 14 consecutive days. The results indicated that the group home residents’ mean PA level on weekdays (7650 ± 2347 steps per day) was below the recommended guidelines from the Hong Kong Medical Association (i.e., 8000 steps per day), and their walk steps were less in the working days as compared to the non-working days of weekends. Another study found that 38 adults with ID working in a sheltered workshop had high percentage of obesity and they were considered as “low active” PA level (5000–7499 steps per day) [20]. However, studies with larger sample size and wider scopes need to be sought to provide more information on PA and PF for this population. Therefore, the purpose of the present paper is to describe the status of PA levels, PF, and sedentary behavior of adults with ID (i.e., differences between sex, ID severity, nonoverweight/obese vs. overweight/obese, low vs. higher risk in central obesity) living in group homes. The secondary purpose is to determine how health-related PF components explain the variance in the PA levels and sedentary behavior of this sampled population.

## 2. Methods

Residents from four selected group homes as convenience samples in Hong Kong that were associated with sheltered workshops (14% of total group homes) were recruited for study. These group homes received Hong Kong government subventions and were managed by non-government organizations. The mean number of residents in the four group homes was 67 adults with ID (standard deviation (SD) = 6.9). Each of the homes had limited indoor space available for physical activities, the smallest having a dining room and an activity room (total area: 135 m^2^) and the largest having a dining room, an activity room, and a dance room (total area: 438 m^2^). Inclusion criteria of study participants were: (i) adult aged 18–65; (ii) diagnosed with either of mild or moderate ID (based on clinical assessment records); and (iii) able to walk without an assisting device. Those eligible were invited to participate and informed consent was obtained from their parents or guardian. Approval for the study was provided by the University’s Human Ethics Committee (FRG2/14-15/062) and the group home managing organizations. Data collection took place between October 2015 and May 2016.

### 2.1. Participant Physical Characteristics

Measures followed the American College of Sports Medicine (ACSM) testing guidelines included body height (assessed by a portable stadiometer, seca gmbh & co. kg., Hamburg, Germany, body weight (TBF-410 Body Composition Analyzer; Tanita Corp., Tokyo, Japan), body mass index (BMI) derived from weight and height, and waist and hip circumferences [21].

### 2.2. Physical Activity Measures

Physical activity levels were assessed using accelerometry (wGT3X-BT Activity Monitor; Actigraph LLC, Pensacola, FL, USA), which has been used extensively in PA studies of adults with ID [22,23,24,25,26,27,28]. In the presence of a test administrator at beginning of each measurement day, the participants put on an elastic belt holding the Actigraph activity monitor around their waists. Group home staff were instructed to remind them to continually wear the monitor during waking hours, with the exception of bath time and bedtime. Participants wore their monitor for at least five consecutive weekdays to a maximum of six weekdays. The monitors were not worn at weekends. Accelerometer data were captured in 60-s periods and non-wear time was defined as ≥60 consecutive minutes of zero recording. To be included in analyses, participants needed four days of accelerometer data with at least 10 h of recording each day [29]. Based on the Freedson et al. cut-offs, moderate-to-vigorous PA (MVPA) was determined at >1951 counts∙min^−1^, while sedentary time was defined as <100 counts∙min^−1^ [30]; any activities in between were classified as light-intensity PA (LPA). Data were summarized as percent of daily time spent in light PA (LPA %), MVPA %, and sedentary behavior (SB %) as well min∙day^−1^ spent in each PA level. 

### 2.3. Health-Related Physical Fitness Measures

Health-related components of PF include body composition, cardiovascular endurance, muscular strength and endurance, and flexibility and measures of health-related PF are closely linked with health promotion and disease prevention [21]. Percent body fat, as one of the indicators of body composition, was assessed by the body fat analyzer (TBF-410 Body Composition Analyzer; Tanita Corp., Tokyo, Japan). Cardiovascular endurance was assessed using the 6-min walk test (6MWT). The 6MWT is a field test for cardiovascular endurance and functional exercise capacity that measures the distance covered when walking on a flat surface (e.g., 20–50 m distance; American Thoracic Society (ATS) Statement 2002 [31]). It has been tested with clinical [31] populations as well as adults with ID [16,32,33,34]. The test has been shown to have both acceptable validity and reliability [32,33,35] for use with adults with ID [32,33,36]. Testing procedures followed the Nasuti et al. [33] study protocol in which each participant walked one trial of walking in laps of a 25-m distance in 6 min provided with regular verbal encouragement prompts and 1:1 pacer. Most studies use a 30-m distance for 6MWT, however, we used 25 m because of available space. We conducted a pilot study with 27 participants tested in 30-m and 25-m distances separately with seven days between and the result showed acceptable intra-class reliability coefficient (R = 0.8) for the 25-m distance. Muscular fitness was assessed using the arm curl test for upper body muscular endurance, which is a fitness test battery for Chinese adults with ID [37] and a test item of the Senior Fitness Test [38,39], previously been shown to be a valid and reliable test with seniors [38,39]. Upper muscular endurance is needed to perform common everyday physical activities that are often difficult to perform in later years. Each participant performed one trial of the arm curl test by having a test administrator counting the completions of the dominant hand in the elbow flexion and extension movement in a 30-s by either moving a 8-lb dumbbell (male) or a 5-lb dumbbell (female) [38]. Lastly, flexibility, being a component of health-related PF, is important to carry out physical activities of daily living [21]. The sit-and-reach test, used to assess the flexibility of trunk flexion has previously been used in studies with people with disabilities [17,37,40,41]. Testing procedure involves two trials of sit-and-reach test with bare feet being placed against a sit-and-reach box with score marked at 23 cm at the level of the feet based on ACSM testing guidelines [21].

### 2.4. Data Analysis

Data were analyzed using IBM SPSS Statistics for Windows, Version 24.0 (IBM Corp., Armonk, NY, USA). Using criteria for Asian adults, participants were categorized into BMI groups (Nonoverweight/Nonobese: BMI < 23; Overweight/Obese: BMI ≥ 23) [11] or groups of low risk vs. higher risk in central obesity based on their waist circumference (low risk: men < 90 cm, woman < 80 cm; higher risk: men ≥ 90 cm, woman ≥ 80 cm) [42]. Independent *t*-tests were computed to determine differences in PA, sedentary behavior, and PF variables between males and females, mild vs. moderate ID, BMI groups, and normal vs. higher risk obesity groups. In view of conducting multiple statistical *t*-tests simultaneously, alpha level was adjusted using the Bonferroni correction. Analysis of covariance was computed to determine differences in PA levels, sedentary behavior, and PF variables between adults with mild ID and moderate ID while controlling for age and sex. Linear multiple regression analysis by two-block entry separately for each dependent variable of PA (LPA %, MVPA %) and sedentary behavior (SB %) was computed. The first block of predictor variables included demographic information (i.e., age, sex, ID type, BMI groups) and all (except age) were inputted by dummy codes. The second block of predictor variables were PF variables including percent body fat, 6MWT, arm curl test, and sit-and-reach test.

## 3. Results

Participants were 71 males and 43 females (36 mild ID, 78 moderate ID) with mean age of 41.7 years (SD = 9.5, range = 18–64 years). The mean number of study participants from the four group homes was 28 (SD = 4.2). PA data from 24 individuals were excluded from analyses (refusal to wear accelerometer, *N* = 5; equipment failure, *N* = 5; <4 valid recording days, *N* = 14), hence, analyses for PA were based on 90 adults. Data analyses for linear multiple regression were based on 81 people with complete dataset (*N* = 6 absent on testing day on 6MWT) and sit-and-reach test (unable to straighten the leg, *N* = 3). Prior to statistical tests, the PA and PF data were checked for normality with MVPA % and MVPA time being two variables having non-normal distribution (MVPA %: skewness = 5.7, kurtosis = 28.0; MVPA time: skewness = 6.1, kurtosis = 42.0). Therefore, natural log transformation for normality on MVPA% and MVPA time was conducted.

Table 1 shows the means and standard deviations of the physical characteristics of the participants as well as PA and PF data. Mean BMI was 24.2 (SD = 5.0) and 55% of the participants (males: 46%, females: 69%; mild ID: 50%; moderate ID: 57%) were overweight or obese (i.e., BMI ≥ 23). Mean waist circumference for males was 83.8 cm (SD = 11.8) and for females was 85.8 (SD = 8.9) and 49% (males: 56%, females: 37%; mild ID: 47%; moderate ID: 50%) were at higher risk in central obesity. Results of anthropometric variables showed that there were significant sex differences in mean scores of height (*p* < 0.001), BMI (*p* = 0.002), hip circumference (*p* < 0.001), and percent body fat (*p* < 0.001) (see Table 1). Furthermore, there was significant difference (*p* = 0.006) in height between adults with mild ID (mean = 1.59 m, SD = 0.12 m) and moderate ID (mean = 1.52 m, SD = 0.10 m).

For the accelerometer data, the average daily wearing time was 726.2 (SD = 96.4 min) for the whole sample (see Table 1). Overall, participants spent their largest percentage of time in sedentary behavior (mean = 67.3%, SD = 12.0%), followed by LPA (mean = 31.1%, SD = 10.8%) and MVPA (mean = 1.6%, SD = 3.4%). Over 80% of the participants (*N* = 74) had no vigorous-intensity PA (VPA). Of those having VPA, they engaged in extremely minimal time (mean = 2 min, SD = 1.8) over the whole data collection period. There were no sex differences in either PA level or in sedentary behavior. For PF variables, with the exception of percent body fat, there were no sex differences in 6MWT, arm curl, and sit-and-reach test. In addition, there were no significant differences in PA levels, sedentary behavior, and PF variables between nonoverweight/nonobese (BMI < 23) and overweight/obese (BMI ≥ 23) participants as well as between those low risk and higher risk in central obesity (based on waist circumference). Similarly, an analysis of covariance by controlling for age and sex showed that there were no mean differences in all PA, sedentary behavior, and PF variables between adults with mild ID and moderate ID (see Table 1).

Regression analyses results showed that the explained variance by R^2^ in the final block separately for LPA %, MVPA %, and SB % were 0.20, 0.36, and 0.24, respectively (see Table 2). Moreover, the significant predictor variable of PF associated with all PA levels and sedentary behavior was 6MWT after controlling for demographic variables. Apart from 6MWT being significant, age was found to be inversely associated with MVPA%. In other words, the older the individual, the less daily time engaged in MVPA.

## 4. Discussion

This study focused on the PA and PF status of adults with ID residing in group homes. Obesity is associated with adverse health outcomes related with metabolic syndrome [42] and half of the participants were overweight or obese (based on BMI data). This is much higher than the general population of HK (39%) [43], consistent with Hong Kong study of 332 adults with ID [44]. This prevalence rate is close to the obesity rates reported of 30% to 50% in this special population in USA [45,46]. Females had higher BMI than males, consistent with Barnes’s findings [23]. In addition to BMI, we used waist circumference as a measure of metabolic and cardiovascular risk. Our results showed that 49% of the participants were at risk, which is higher than the 32% of adults at risk in the general population in Hong Kong [47]. Previous findings have suggested obesity rates for those adults with ID living in restrictive environments such as group homes are higher than those living in institutional or natural family groups [46], which may explain the higher percentage of overweight or obese and central obesity risk in this study.

We found no sex differences and no differences between ID severity groups in terms of PA levels, which are contradictory to other studies that have shown males with ID to be more active than their counterparts [48,49] and adults with mild ID to be more active than those with moderate ID [50,51]. We found no differences in PA levels and sedentary behavior between nonoverweight/nonobese vs. overweight/obese groups. This contrasts with Barnes et al. [23] who found differences time spent in MVPA by BMI status (BMI < 25 vs. BMI ≥ 25) in adults with ID. The Barnes study, however, included adults with ID living in diversified settings while all our participants lived in group homes. Previous research has shown that living in common care settings and not having daytime PA opportunities were independent predictors of low PA [48]. Our participants were all residents of group homes that had limited indoor activity space and all attend sheltered workshops during weekdays (9:00 a.m. to 4:30 p.m.) where most of their work was done while sitting. Living in similar, restrictive environments may explain our finding of no sex or severity of ID differences in PA levels because all residents share similar daily lifestyle patterns.

Our study found that, on average, the participants spent little time in MVPA (10 min daily), lower than that reported for adults with ID in previous studies (from 13 to 36 min∙day^−1^) [24,52,53] and over 80% of them had no VPA. Although of similar mean age (44 years old), participants in Oviedo’s study did not reside in group homes and the study used different cut-off criteria for MVPA [52]. The very low MVPA% in our study could possibly be due to most residents walking only a very short distance to the sheltered workshop that was in the same building or because measurements were taken only on weekdays. It was not known whether adults with ID would engage in more PA during weekends particularly for those spending weekends away from their group homes. We have no data on how active the participants were when away from the group homes on weekends. Nevertheless, having an average of 10 min of MVPA per weekday suggests the participants will fall far short of the WHO recommendation of accumulating at least 150 MVPA min of MVPA in a week.

The participants spent 67.3% of awake time in sedentary behavior (495 out of 726 min∙day^−1^) and they are mostly sitting during work in the sheltered workshop, at meal-times, and in leisure time after dinner. Overall, this amount was lower than the 522 min to 643 min assessed by objective measures in other studies of adults with ID [54]. Nonetheless, the proportion of time participants were sedentary when wearing accelerometers (67.3%) was within the range of 63.0 to 87.5% reported by a review paper on sedentary behavior in adults with ID [54]. In particular, this result was identical to those two studies (63–64%) that also adopted Freedson’s cut-off point for sedentary behavior (<100 counts∙min^−1^) [22,55]. When comparing time in sedentary behavior with USA National Health and Nutrition Examination Survey (NHANES) data involving adults without ID, participants in this study sample had a slightly higher sedentary time than those without ID (495 min vs. 479 min), however, NHANES data had 14 h of accelerometer wear time or 2 h more than the present study [56]. This high proportion of being sedentary is substantiated by anecdotal accounts by group home staff that the majority of residents were not interested in engaging in physical activity or exercise during their leisure time.

The health-related components of PF have a strong relationship with overall health including a lower prevalence of chronic diseases [57]. The participants with ID performed much lower on the health-related PF components than their counterparts without ID did in previous studies [21,58]. Additionally, on the 6MWT (walking capacity) the participants scored lower than adults with ID in Nordstrøm’s study [22] (434 m vs. 481 m). This difference could perhaps be explained by the participants on our study being older (18–64 years vs. 16–45 years). Results for the arm curl test were similar to another study from Hong Kong in 166 adults with ID [37] but was at the 25th percentile for older (65–69 years) Hong Kong people without ID [58]. The study participants are inflexible in trunk flexion and their mean value is comparable to the lowest category of “needs improvement” of 40–49 age group adults from the general population [21]. Results of poorer cardiovascular fitness, upper body muscular endurance, and trunk flexion flexibility may affect the participants’ ability to perform certain physical activities in daily living.

In the determination of PF variables contributing to the percentage of time spent in PA levels and sedentary behavior, the 6MWT test measuring cardiovascular fitness was found to be the only significant predictor of time spent in PA levels and sedentary behavior. Cardiovascular fitness has an inverse relationship with risk of all cause premature death [59,60] and higher levels of cardiovascular fitness are associated with higher levels of habitual physical activity in the general population [61]. The results from the multiple regression analyses were consistent with Williams’ conclusion [61] of the direct positive relationship between 6MWT (cardiovascular fitness) and more time spent in habitual daily PA. Walking has been shown as the most common PA engaged in across all levels of ID [23,48]. However, adults with ID do not habitually walk fast or for long periods of time [48]. Encouraging and providing support for adults with ID to walk more has potential as an intervention strategy. A multi-component weight loss intervention study with adults with ID found that providing targets of daily steps and pedometers significantly increased LPA and decreased sedentary behavior [54].

In view of our current findings of low PA and low fitness levels in this population subgroup, intervention studies can set goals to increase both PA and PF for adults with ID. Indeed, interventions targeting PA and PF found significant improvements for PA and for fitness outcomes such as aerobic fitness and muscular strength [62,63] in adults with ID. Considering the study participants being residents in group home setting where they spend most of the day time in sitting, interventions for PA might target lower intensity PA in which adults with ID seem to have a higher preference over MVPA [4]. Although the WHO’s PA recommendation focuses on 150 min of at least moderate intensity PA per week, recent meta-analytical study has concluded that LPA could improve adult cardio-metabolic health and reduce mortality risk [64]. Emerging evidence suggests that LPA can convey health benefits and that starting to participate in PA of lower than moderate-intensity could yield positive health benefits for inactive individuals [65,66]. Nonetheless, it is still not known how well adults with ID may adhere to either low-or moderate-intensity PA programs. Further study is needed to determine the intervention efficacy of different intensity levels for this subgroup.

A strength of this study is its assessment of PA using accelerometry; nonetheless, it has some limitations. Participants were a convenience sample from four group home locations in HK. PA data were limited to weekdays and about half the participants did not stay at the group homes during weekends meaning their PA could have varied on these days. The Freedson cut points for PA levels were used in the study as in a previous study among adults with ID [23]; however, there is no consensus on accelerometer cut-off criteria. During testing a few younger participants (e.g., age around 20) inquired about running rather than walking for the 6MWT, so future studies involving younger adults might consider a field test requiring higher intensity (e.g., shuttle run test) [54] for assessing cardiovascular fitness. The implications for this study are that for (i) administrators and practitioners working in care settings for people with ID, they should be made aware of low PA and PF and employ effective strategies to address this and for (ii) researchers, future studies are needed to further investigate the activity patterns of adults with ID during weekends as well as to determine if there will be differences in their activity patters between those living in group homes of different physical sizes.

## 5. Conclusions

This study provides PA and fitness profiles of a sample of adults with ID residing in group homes. It shows that adults with ID are extremely sedentary in their homes during weekdays and they have lower fitness levels compared with previous studies of adults without disabilities. PA and low fitness levels could be attributed to the lifestyle including a seated job environment at a sheltered workshop, a relatively restrictive living environment residing in a group home, and the close proximity of the work place to the group home. Future PA interventions are needed to target for effective strategies such as PA promotion during breaks at work and leisure time after work that can increase time for LPA and MVPA and decrease time for sedentary behavior for adults with ID. Furthermore, future study should determine whether intervention program targeting at increasing physical fitness of adults with ID can also improve their habitual PA levels.

## Figures and Tables

**Table 1:** Physical activity and physical fitness of adults with intellectual disabilities (ID) overall, and by sex and severity of intellectual disabilities.

	**All**	**Sex**		**Type of ID**			
**Physical Characteristics**	***N* = 114**	**Males** ***N* = 71**	**Female** ***N* = 43**	***p*-Values**	**Mild** ***N* = 36**	**Moderate** ***N* = 78**	***p*-Values**
Age	41.7 (9.5)	40.7 (9.7)	43.3 (9.1)	0.164	40.7 (8.3)	42.1 (10.1)	0.446
Height (cm)	1.5 (0.1)	1.6 (0.1)	1.5 (0.1)	<0.001 *	1.6 (0.1)	1.5 (0.1)	0.006 *
Weight (kg)	57.8 (11.7)	57.7 (13.1)	57.9 (8.9)	0.903	60.1 (13.7)	56.6 (10.5)	0.139
BMI (kg/m^2^)	24.2 (5.0)	23.3 (5.0)	26.3 (4.5)	0.002 *	23.8 (4.3)	24.7 (5.3)	0.387
BMI < 23 (%)	45	54	31		50	43	
BMI ≥ 23 (%)	55	46	69		50	57	
WC (cm)	84.6 (10.8)	83.8 (11.8)	85.8 (8.9)	0.360	85.7 (12.1)	84.1 (10.3)	0.463
Low Risk (%)	51	44	63		53	50	
Higher Risk (%)	49	56	37		47	50	
HC (cm)	95.0 (9.4)	92.0 (9.2)	99.9 (7.3)	<0.001 *	94.5 (8.7)	95.2 (9.7)	0.730
**PA Variables**	***N* = 90**	**Males** ***N* = 56**	**Female** ***N* = 34**	***p*-Values**	**Mild** ***N* = 29**	**Moderate** ***N* = 61**	***p*-Values ^a^**
LPA %	31.1 (10.8)	31.8 (11.1)	29.9 (10.2)	0.414	30.6 (9.9)	31.3 (11.2)	0.674
MVPA %	1.6 (3.4)	1.6 (3.1)	1.5 (3.9)	0.904	0.9 (0.7)	1.9 (4.0)	0.216
SB %	67.3 (12.0)	66.6 (12.6)	68.6 (11.0)	0.445	68.5 (10.4)	66.8 (12.8)	0.429
LPA time (min∙day^−1^)	229.9 (85.3)	235.3 (89.4)	221.0 (78.6)	0.443	225.7 (75.0)	232.0 (90.3)	0.950
MVPA time (min∙day^−1^)	9.9 (19.7)	12.2 (24.4)	6.2 (5.1)	0.168	7.1 (5.2)	11.3 (23.6)	0.757
SB time (min∙day^−1^)	495.4 (87.1)	488.4 (92.0)	506.9 (78.3)	0.333	503.4 (84.7)	491.6 (88.7)	0.645
Wearing time (min∙day^−1^)	726.2 (96.4)	721.3 (112.5)	734.1 (62.4)	0.546	736.2 (59.4)	721.4 (109.9)	0.425
**Physical Fitness**	***N* = 114**	**Males** ***N* = 71**	**Female** ***N* = 43**	***p*-Values**	**Mild** ***N* = 29**	**Moderate** ***N* = 61**	***p*-Values ^a^**
% Body fat	26.0 (11.6)	19.3 (7.9)	37.2 (7.4)	<0.001	25.7 (10.6)	26.2 (12.1)	0.439
6MWT (m)	434.1 (106.5)	444.0 (117.9)	418.7 (85.1)	0.231	446.0 (124.6)	428.6 (97.4)	0.475
Arm curl (reps.)	11.4 (4.3)	11.7 (4.7)	11.0 (3.8)	0.379	12.5 (12.2)	11.0 (4.2)	0.069
Sit and reach (cm)	14.9 (13.6)	14.4 (14.2)	15.8 (12.8)	0.587	(12.2)	15.2 (14.3)	0.818

Note: * *p* < 0.05. ^a^ Statistical tests between those with mild ID and moderate ID for physical activity (PA) levels, sedentary behavior, and physical fitness variables were adjusted for age and sex. Abbreviations: 6MWT, six-minute walk test; BMI, body mass index; HC, hip circumference; LPA, light-intensity physical activity; MVPA, moderate-to-vigorous physical activity; SB, sedentary behavior; WC, waist circumference.

**Table 2:** Contribution of physical fitness variables in explaining variance of physical activity and sedentary behavior

**LPA % (R^2^ = 0.199)**	**β**	**95% CI**	**t**	***p-*Values**
% Body fat	−0.049	−0.529 to 0.433	−0.199	0.843
6MWT	0.417	0.016 to 0.072	3.105	0.003 *
Arm curl	−0.067	−0.780 to 0.453	−0.528	0.599
Sit and reach	−0.092	−0.272 to 0.119	−0.776	0.440
**MVPA % (R^2^ = 0.360)**				
Age ^a^	−0.426	−0.071 to −0.026	−4.320	<0.001 *
% Body fat	0.068	−0.036 to 0.049	0.308	0.759
6MWT	0.361	0.001 to 0.006	3.008	0.004 *
Arm curl	−0.133	−0.087 to 0.022	−1.181	0.241
Sit and reach	0.119	−0.008 to 0.027	1.132	0.262
**SB % (R^2^ = 0.240)**				
% Body fat	0.114	−0.651 to 0.401	−0.474	0.637
6MWT	−0.416	−0.080 to −0.018	−3.182	0.002 *
Arm curl	0.083	−0.446 to 0.902	0.675	0.502
Sit and reach	0.037	−0.179 to 0.248	0.322	0.748

Note: * *p* < 0.05. ^a^ Regression models adjusted by demographical variables of age, sex, body mass index groups. Abbreviation: CI, confidence interval.
